# Integrated Analysis of Genetic, Spectral, Phenotypic, and Stress-Resistant Traits in *Vanda* × *Papilionanthe* Intergeneric Hybrids

**DOI:** 10.3390/plants15071083

**Published:** 2026-04-01

**Authors:** Huan Li, Xue-Qiang Cui, Zi-Bin Zhang, Jia-Wei Li

**Affiliations:** 1 Guangxi Key Laboratory of Forest Ecology and Conservation, School of Forestry, Guangxi University, Nanning 530004, China; li1409128226@163.com; 2 Flower Research Institute, Guangxi Academy of Agricultural Sciences, Nanning 530007, China; yncuixueqiang@126.com (X.-Q.C.); candou154@126.com (Z.-B.Z.)

**Keywords:** water potential at turgor loss point, intergeneric hybridization, genetic diversity, spectral phenotype, floral water balance, high-temperature tolerance

## Abstract

Intergeneric hybridization of *Vanda* and *Papilionanthe* holds promise for pyramiding superior ornamental and stress-tolerant traits, though systematic studies on their hybrids remain scarce. Using *Vanda lamellata* var. *Boxallii* (♀), *Papilionanda* ‘Hetty Henderson’ (♂), and 72 progenies, we investigated parent–progeny relationships via iPBS markers, spectral phenomics, and morphology, alongside floral water balance and thermotolerance. Six iPBS primers amplified 90 bands (92.98% polymorphism), confirming high genetic diversity. Spectral reflectance (400–1000 nm) revealed organ-specific genetic differentiation. Clustering analyses consistently indicated that progenies were genetically and phenotypically closer to the female parent, with spectral/morphological patterns matching genetic groupings. Resistance evaluations showed progenies had significantly stronger floral water storage capacity than both parents, while the female parent excelled in water transport traits. Progenies developed thicker petal/sepal cuticles, though the male parent exhibited superior thermotolerance indices. This study clarifies the genetic regulation of stress resistance in these hybrids, providing critical support for precise early screening in orchid breeding.

## 1. Introduction

*Vanda* is a genus of high horticultural and ornamental value, mainly distributed in tropical regions, including Southeast Asia, South Asia, and northern Australia. Characterized by its unique floral morphology, rich flower color variations, erect inflorescences, long flowering duration, and strong environmental adaptability, it therefore occupies an important role in the industrial development of tropical orchids [1,2,3,4,5]. *Papilionanthe* is a genus segregated from the genus *Vanda*. Molecular phylogenetic evidence derived from multiple molecular markers—including random amplified polymorphic DNA (RAPD) markers, nuclear ribosomal DNA internal transcribed spacer (ITS) sequences, and chloroplast gene fragments—has demonstrated substantial evolutionary divergence between *Papilionanthe* and *Vanda* [3,6,7]. More recently, the application of inter-primer binding site (iPBS) markers has further corroborated this evolutionary distinction [8], providing robust molecular support for research on the genetic differentiation between the two genera. Owing to its distinctive petal texture and elite stress-resistance genes, *Papilionanthe* represents a highly promising genetic resource for orchid breeding. Intergeneric hybridization between *Vanda* and *Papilionanthe* represents a highly valuable strategy in orchid breeding, with a documented history tracing back to the mid-20th century. This breeding approach is designed to pyramid elite horticultural and adaptive traits from both parental genera, including the large flower size and vibrant floral pigmentation of *Vanda*, as well as the robust stress tolerance and distinctive floral morphology of *Papilionanthe* [9]. Nevertheless, comprehensive studies focusing on the genetic diversity, morphological variation, and eco-physiological adaptability of *Vanda* × *Papilionanthe* intergeneric hybrids remain largely insufficient.

Molecular markers are important tools for evaluating genetic diversity and genetic relationships in *Vanda* and its hybrids. For example, inter-simple sequence repeat (ISSR) markers have been used to analyze the genetic diversity of parental species such as *Vanda dearei* and *V. celebica* and their hybrid progeny [10]. Two single primer amplification reaction (SPAR) methods, namely random amplified polymorphic DNA (RAPD) and inter-simple sequence repeat (ISSR), have been applied to assess genetic variation in wild *Vanda* genotypes [11]. Among various molecular markers, the iPBS marker amplifies regions targeting the two primer binding site (PBS) regions in long terminal repeat (LTR) retrotransposons [12]. Because LTR retrotransposons are widely distributed in nature, and iPBS markers feature low technical difficulty, simple operation, high reliability, low cost, and universal primers that require no preliminary design or screening, this technique has been widely applied in many fields. iPBS markers have also been used to analyze genetic diversity and phylogenetic relationships among 36 species of *Vanda* and two species of *Aerides*, and to establish DNA fingerprints [8]. These studies provide valuable references for determining the genetic diversity and genetic relationships of intergeneric hybrids between *Vanda* and *Papilionanthe*.

Morphological traits are the most intuitive phenotypic characteristics of plants and core indicators in traditional breeding research [13,14]. However, morphological traits exhibit strong phenotypic plasticity due to the influence of environmental conditions, developmental stages and cultivation management practices [15,16]; relying solely on morphological markers not only makes it difficult to accurately reflect the actual genetic differences but also leads to subjectivity in identification results, whereas molecular markers can directly reveal genetic variation at the genomic level with high stability and without environmental interference, allowing for an objective assessment of the genetic relationships among germplasm accessions. For instance, Zhao et al. [17] used iPBS markers combined with morphological markers to identify *Dendrobium* hybrids, confirming that iPBS markers can efficiently identify *Dendrobium* hybrids, while morphological markers facilitate the screening of progeny with elite traits. Spectroscopic techniques capture the absorption, reflection, and transmission characteristics of plants across different spectral bands, thereby indirectly reflecting differences in internal physiological metabolism and external morphological structure [18,19]. Tepal spectral phenotypic data obtained by portable spectrometers have been applied in various studies on floral organs due to their rapid, non-destructive, and high-throughput advantages. For example, hyperspectral imaging has been used for non-destructive and efficient determination of flower color phenotypes, pigment composition, and content in different parts of chrysanthemum cultivars [20]. Researchers non-destructively determined the absorption characteristics of *Petunia* petals using light scattering spectroscopy and clarified pigment distribution patterns combined with microstructures [21]. Wang et al. [22] measured the reflectance spectra of floral organs in male and female flowers of *Ottelia cordata* and found that female flowers exhibited significantly higher reflectance in the UV-blue band than male flowers. However, relying solely on spectral data only reflects physicochemical differences in phenotypes and fails to reveal patterns of genetic differentiation; using molecular markers alone makes it difficult to directly link to ornamental trait performance; and depending only on morphological observations is prone to environmental interference. At present, studies that integrate morphological markers, molecular genetic markers, and spectral phenotyping techniques to systematically analyze the genetic relationships and diversity of hybrid progeny remain scarce, which hinders the comprehensive and objective evaluation of the overall genetic characteristics of hybrid germplasm.

Whether hybrid progeny exhibit generally stronger advantages in water and high-temperature adaptation depends on the combined effects of specific cross combinations, genetic backgrounds, and environmental conditions [23,24]. Many studies have shown that hybrid progeny may indeed display heterosis [25,26]. Nevertheless, such advantages are not universal; sometimes hybrids show reduced adaptability or intermediate performance relative to the parents [27]. In orchid research, studies on the morphological and physiological responses of *Vanilla planifolia*, *V. pompona*, and their hybrids to water stress have demonstrated that hybrid progeny have potential application advantages under the global drought trend [28]. Another study compared the morphological and physiological performances of parental lines *Epidendrum orchidiflorum* and *E. denticulatum* and their hybrids under different water deficit conditions, revealing that the hybrids were significantly less tolerant to water stress than the parents [27]. Thus, evaluating stress resistance in hybrid progeny based solely on physiological phenotypes is limited and insufficient to explain the genetic basis underlying such differences.

In this study, *Vanda lamellata* var. *Boxallii* Rchb. (female) and *Papilionanda* ‘Hetty Henderson’ were used to obtain intergeneric hybrids between *Vanda* and *Papilionanthe*. We systematically analyzed parent–progeny relationships at the molecular, spectral phenotypic, and morphological levels, to reveal the genetic basis of hybrid traits and support early targeted trait screening in breeding. We further compared floral organ water balance strategies and high-temperature tolerance between parents and progeny, to characterize the adaptive performance of hybrid progeny at the ornamental organ level.

## 2. Results

### 2.1. Genetic Diversity Analysis

**Figure 1:**
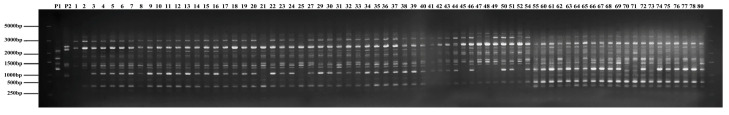
Agarose gel electrophoresis of genomic DNA amplified by 2232 primer pairs in parental lines and 72 progeny germplasm resources.

**Figure 2:**
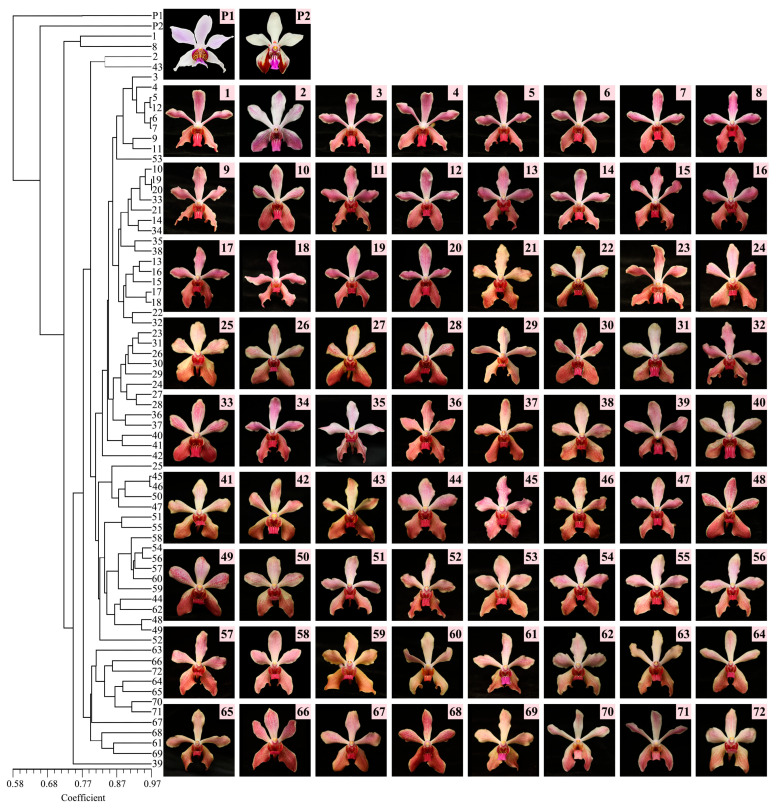
UPGMA cluster analysis of parental lines and 72 progeny germplasm resources, with a legend illustrating the relationship between parents and progeny.

Six primers with clear amplification bands, high polymorphism, and good repeatability were screened from 83 iPBS primers. These primers were used for PCR amplification of genomic DNA from the *Vanda* parental lines and 72 hybrid progeny lines. The amplification results (Table 1) showed that a total of 90 bands were amplified by the six primers, of which 84 were polymorphic, with an average polymorphism ratio of 92.98% and an average of 15 bands per primer. Primers including iPBS-2232 and iPBS-2251 exhibited 100% polymorphism, which was significantly higher than that of other primers. The lowest polymorphism rate (85.71%) was observed for primer iPBS-2270. Primer iPBS-2232 showed the best amplification efficiency, generating the largest number of bands (19), with a polymorphic percentage locus (PPL) of 100% (Figure 1). Genetic diversity indices of the *Vanda* parental lines and 72 progeny accessions were calculated using POPGENE 32 software (Table 2). The mean observed number of alleles (Na) was 1.933, the mean effective number of alleles (Ne) was 1.489, the mean Nei’s genetic diversity (He) was 0.293, and the mean Shannon’s information diversity index (I) was 0.443. These results indicated substantial genetic variation among the 74 germplasm accessions, confirming that *Vanda* possesses abundant genetic diversity. Based on the iPBS amplification profiles, the Dice genetic similarity (GS) coefficient matrix was computed using the SIMQUAL module in NTSYS-pc 2.10e software, and cluster analysis was performed on the 74 accessions using the unweighted pair-group method with arithmetic mean (UPGMA) (Figure 2).

### 2.2. Spectral Characteristics and Ornamental Trait Analysis of Parental Lines and Hybrid Progeny

**Figure 3:**
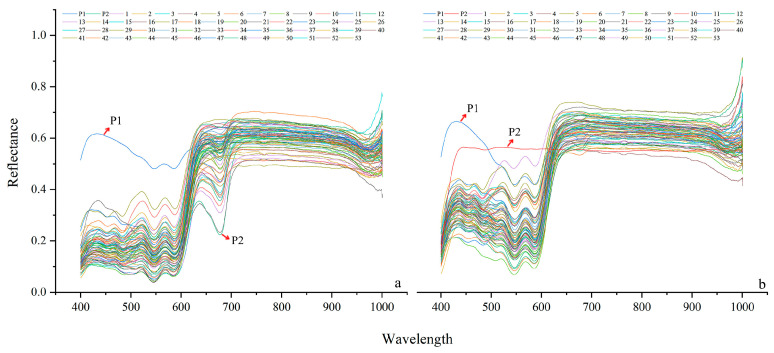
Reflectance spectra of floral organs in parental lines and progeny. Spectral reflectance (Reflectance, 0–1.0) curves of sepals (**a**) and petals (**b**) as a function of wavelength (Wavelength, nm), representing the reflectance capacity of floral organs to light of different wavebands.

The spectral characteristics of sepals exhibited a different inheritance pattern. Within the 400–700 nm characteristic band, the overall trends of the spectral curves of the female parent and hybrid progeny were highly consistent. The male parent only coincided with the hybrid progeny at two characteristic points: 550 nm (valley) and 570 nm (peak). The sepal spectra of all parents and hybrid progeny stabilized after 700 nm, and sepals displayed an additional characteristic valley at 650–700 nm compared with petals (Figure 3a). Petal spectral analysis showed that all progeny lines exhibited consistent overall trends in spectral curves, and the genetic effects between parents and hybrid progeny displayed obvious band specificity. The male parent and hybrid progeny showed consistent peak and valley characteristics in the 500–600 nm band. However, in the 400–500 nm band, the spectral characteristics of the male parent and hybrid progeny differed significantly. The female parent and hybrid progeny showed no obvious differentiation in spectral trends within the 400–600 nm characteristic band, and their spectral curves stabilized after 450 nm. In contrast, the spectral curves of the male parent and hybrid progeny did not stabilize until after 650 nm (Figure 3b).

**Figure 4:**
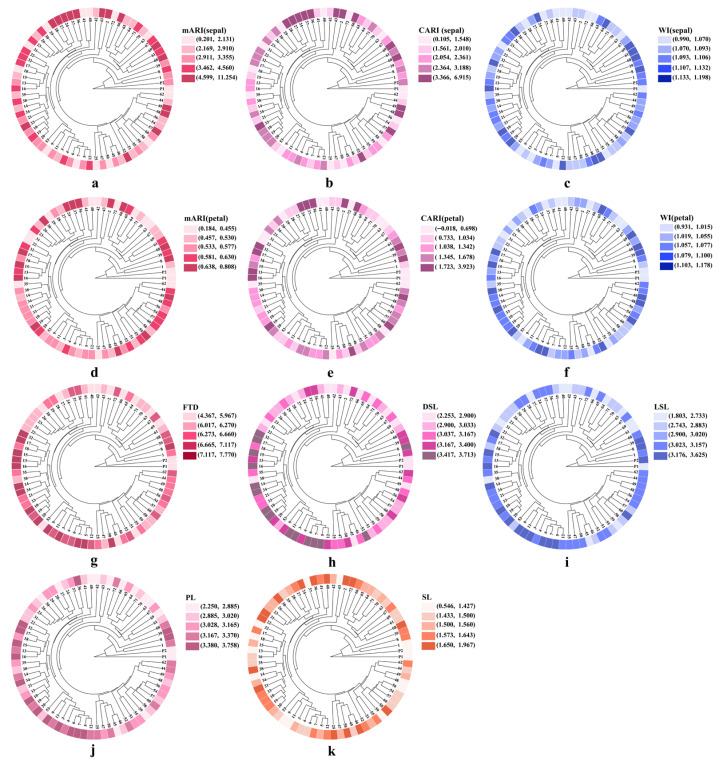
Circular phylogenetic clustering tree of 72 *Vanda* progeny and parental lines based on iPBS molecular markers, integrated with the numerical gradient distributions of phenotypic indices. (**a**) modified anthocyanin reflectance index (MARI) of sepals; (**b**) carotenoid reflectance index (CARI) of sepals; (**c**) water index (WI) of sepals; (**d**) MARI of petals; (**e**) CARI of petals; (**f**) WI of petals; and clustered based on five flower morphological traits with heterosis, including (**g**) flower transverse diameter (FTD); (**h**) dorsal sepal length (DSL); (**i**) lateral sepal length (LSL); (**j**) petal length (PL); (**k**) spur length (SL). Peripheral colored bars represent graded values of each index. Taking MARI in panel (**a**) as an example, the color gradient from light pink to dark red corresponds to values ranging from 0.184 to 0.809, and the color gradients for the other indices correspond to their respective numerical ranges.

The modified anthocyanin reflectance index (MARI) and carotenoid reflectance index (CARI) reflect the relative accumulation of anthocyanins and carotenoids in floral organs, respectively. The results (Figure 4a–f, Appendix A Table A1) showed that the ranges of MARI (0.184–0.808) and CARI (−0.018–3.923) in petals were significantly lower than those in sepals (0.201–11.254; 0.105–6.915). The petal water index (WI) values of both parents were relatively low (0.931–1.055, light color), and most progeny lines had higher values than the parents (P1 = 1.013, P2 = 0.990). For sepal WI, all progeny lines had higher values than the male parent (P1 = 1.023), while the female parent (P2 = 1.119) showed a high value range (1.107–1.198, dark color), higher than most progeny lines.

**Figure A1:**
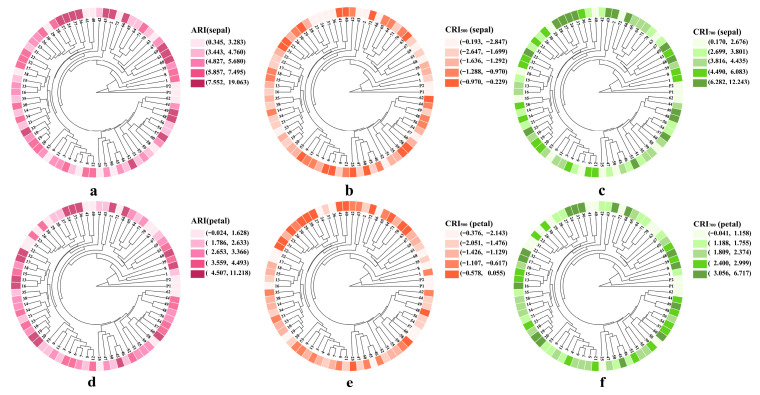
Circular phylogenetic clustering tree of 72 *Vanda* progeny and parental lines based on iPBS molecular markers, integrated with the numerical gradient distributions of phenotypic indices. (**a**) anthocyanin reflectance index (ARI) of sepals. (**b**) carotenoid reflectance index (CRI_500_) of sepals. (**c**) carotenoid reflectance index (CRI_700_) of sepals. (**d**) anthocyanin reflectance index (ARI) of petals. (**e**) carotenoid reflectance index (CRI_500_) of petals. (**f**) carotenoid reflectance index (CRI_700_) of petals.

Within the same clade, MARI and CARI distribution patterns in petals were largely consistent. In sepals, only a few samples showed low index values: samples with low MARI values were 25 (1.596), 50 (1.823), 51 (2.386), 58 (1.018), and 62 (1.527); samples with low CARI values were 25 (1.254), 50 (1.370), 46 (1.942), 58 (0.706), and 62 (1.064). The distribution patterns of WI in petals and sepals were also generally consistent. The clustering distribution of these phenotypic indices was highly consistent with the genetic clades defined by iPBS molecular markers, indicating that the phenotypic variation of floral spectral indices in *Vanda* was related to the genetic background revealed by iPBS markers. Further analysis of the anthocyanin reflectance index (ARI) and carotenoid reflectance indices (CRI_500_ and CRI_700_) in sepals and petals revealed similar variation trends to those of the above spectral indices, with slight differences in characterization (Appendix B Figure A1).

The clustering distribution of different floral organ morphological indices is shown in Figure 4g–k. High values of flower transverse diameter (FTD, 6.273–7.770, dark color), dorsal sepal length (DSL, 3.037–3.713, dark color), lateral sepal length (LSL, 3.023–3.625, dark color), and petal length (PL, 3.028–3.758, dark color) were mainly distributed in the lower-left region of the dendrogram and clustered into the same clade. A small number of samples in this clade showed low trait values: samples 22, 38, and 53 exhibited light colors (low values) for all four indices (FTD, DSL, LSL, PL). In addition, sample 18 in DSL and sample 21 in PL also belonged to the light-color (low-value) group. The high-value traits of FTD, DSL, LSL, and PL showed strong clustering synergy and were mainly concentrated in specific genetic clades, with only a few samples showing phenotypic differentiation in some indices. This provides a phenotypic basis for further analyzing the genetic linkage of floral organ morphological traits. For the spur length (SL) trait, among samples 36, 37, 24, 27, 28, 29, 30, 26, 23, and 31, only 37 and 29 showed light colors (low values). Samples with high spur length (1.659–1.967, dark color) were locally clustered in the genetic dendrogram, mainly distributed in the marginal clades. In contrast, samples with low values (0.546–1.427, light color) were scattered across multiple clades without obvious clustering preference.

The flower size of the male parent was larger than that of the female parent. The hybrid progeny were more similar to the male parent in flower size, but closer to the female parent in flower morphology (Table 3). The measured traits included flower transverse diameter (FTD), dorsal sepal length (DSL), dorsal sepal width (DSW), petal length (PL), petal width (PW), lateral sepal length (LSL), lateral sepal width (LSW), labellum length (LL), labellum width (LW), spur length (SL), pedicel length (PedL), inflorescence length (IL), leaf length (LEL), and leaf width (LEW).

**Figure A2:**
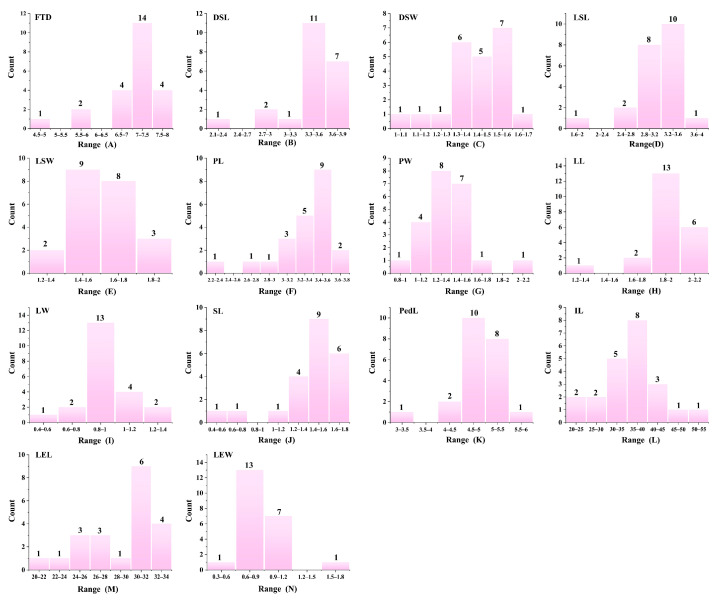
Frequency histograms of 14 phenotypic traits in 20 hybrid progeny of *Vanda*. The *x*-axis represents the phenotypic value ranges (**A**–**N**) of each trait, and the *y*-axis shows the count (frequency) of progeny individuals falling within each range. The traits include flower transverse diameter (FTD), dorsal sepal length (DSL), dorsal sepal width (DSW), lateral sepal length (LSL), lateral sepal width (LSW), petal length (PL), petal width (PW), labellum length (LL), labellum width (LW), spur length (SL), pedicel length (PedL), inflorescence length (IL), leaf length (LEL), and leaf width (LEW).

For flower-size-related traits, the mean FTD and DSL in the progeny were 7.13 cm and 3.45 cm, respectively, with 95.00% of the progeny exceeding the higher parental values; the mean values of LSL, PL, SL, and LEL were 3.40 cm, 3.20 cm, 1.49 cm, and 29.68 cm, respectively, with 100% of the progeny exceeding the parental values. In terms of trait variation, PW, LSW, LW, and IL exhibited relatively high coefficients of variation (12.30%, 9.83%, 13.75%, and 18.12%, respectively), while all progeny showed intermediate values between the two parents for DSW, PW, LL, LW, and LEW. Frequency histograms were plotted to describe the distributions of the 14 phenotypic traits in two parents and 20 progeny individuals (Appendix B Figure A2). Most traits showed continuous variation, indicating typical quantitative inheritance. PedL and LW had narrow distributions, with the highest frequencies (18 and 13 counts, respectively) falling within 4.5–5.5 cm and 0.6–0.8 cm, reflecting concentrated phenotypic variation.

### 2.3. Differences in Water Balance Strategies

**Figure 5:**
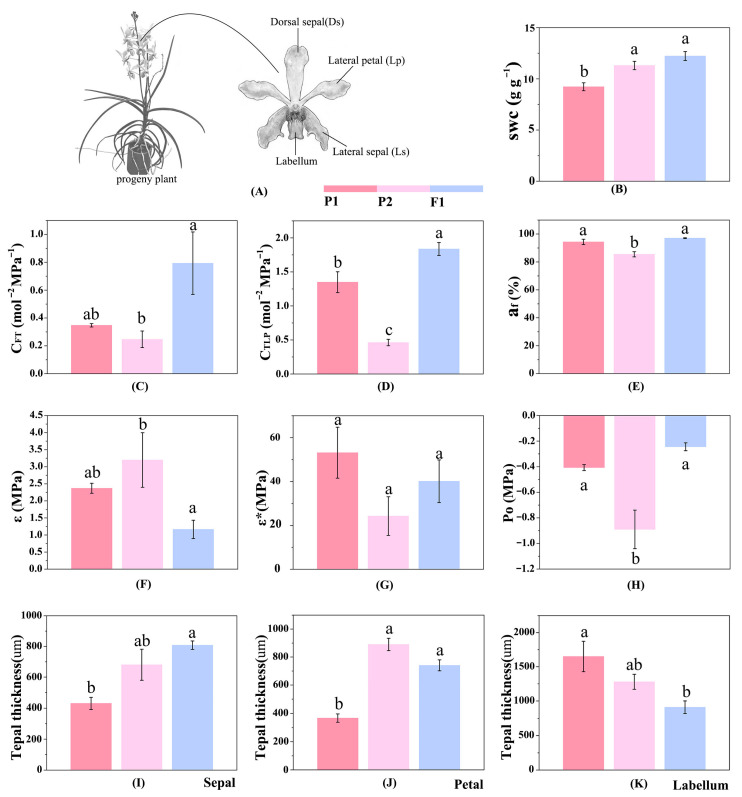
Analysis of differences in water balance capacity between parental lines and progeny. (**A**) Schematic representation of flower size and floral structure in progeny. (**B**) Saturated water content (SWC, g g^−1^). (**C**) Hydraulic capacitance of floral tissue before turgor loss point (C_FT_, mol m^−2^ MPa^−1^). (**D**) Hydraulic capacitance at turgor loss point (C_TLP_, mol m^−2^ MPa^−1^). (**E**) Apoplastic water fraction (a_f_, %). (**F**) Bulk modulus of elasticity ε (MPa). (**G**) Normalized bulk modulus of elasticity ε* (MPa). (**H**) Saturation turgor pressure P_0_ (MPa). (**I**) Sepal thickness (μm); (**J**) Petal thickness (μm). (**K**) Labellum thickness (μm). All data are presented as means ± SD. Different letters above bars within the same floral organ indicate significant differences between treatments (*p* < 0.05, based on one-way ANOVA followed by Tukey’s post hoc test).

Floral organs of the male parent, female parent, and progeny showed significant differences in water balance-related traits to varying degrees (Figure 5). For water storage capacity-related traits, petal and sepal thickness of progeny showed no significant difference from the female parent, but were significantly higher than those of the male parent (*p* = 0.010, *p* = 0.014). Labellum thickness of progeny was significantly lower than that of the male parent (*p* = 0.033). Saturated water content (SWC) of progeny showed no significant difference from the female parent, but was significantly higher than that of the male parent (*p* = 0.006). The apoplastic water fraction (a_f_) showed no significant difference from the male parent, but was significantly higher than that of the female parent (*p* = 0.005). Hydraulic capacitance before turgor loss (C_FT_) showed no significant difference from the male parent, but was significantly higher than that of the female parent (*p* = 0.012). Hydraulic capacitance at turgor loss point (C_TLP_) differed significantly from both parents and was significantly higher than that of both parents (*p* = 0.018, *p* < 0.001).

**Figure A3:**
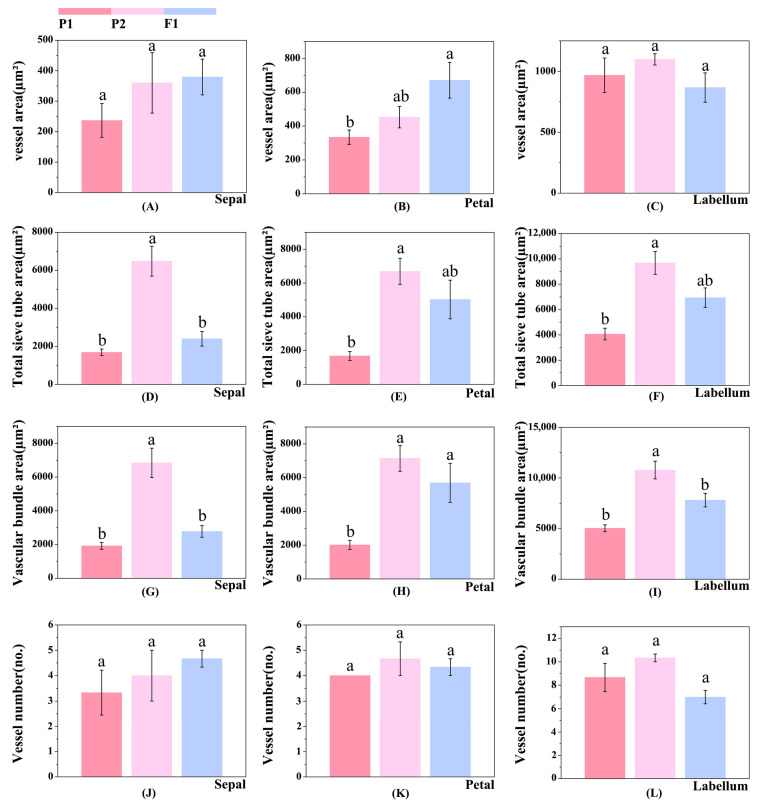
Water supply-related traits in parents and progeny. (**A**) Vessel area of sepals (µm^2^). (**B**) Vessel area of petals (µm^2^). (**C**) Vessel area of labellum (µm^2^). (**D**) Total sieve tube area of sepals (µm^2^). (**E**) Total sieve tube area of petals (µm^2^). (**F**) Total sieve tube area of labellum (µm^2^). (**G**) Vascular bundle area of sepals (µm^2^). (**H**) Vascular bundle area of petals (µm^2^). (**I**) Vascular bundle area of labellum (µm^2^). (**J**) Vessel number of sepals (number). (**K**) Vessel number of petals (number). (**L**) Vessel number of labellum (number). All data are presented as means ± SD. Different letters above bars within the same floral organ indicate significant differences among treatments (based on one-way ANOVA followed by Tukey’s post hoc test).

**Figure A4:**
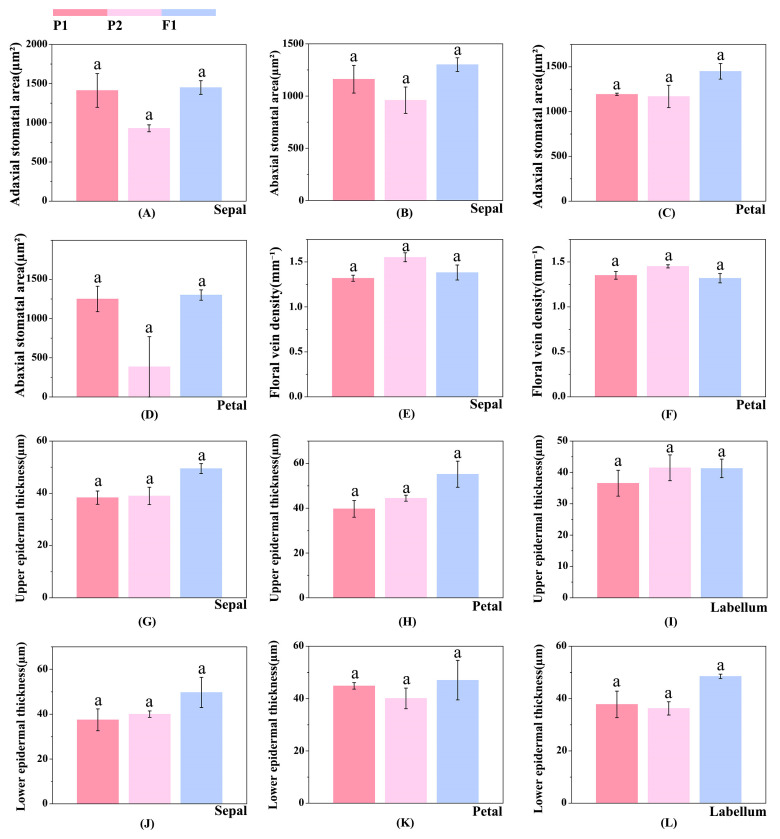
Structural traits of floral organs in parents and progeny. (**A**) Stomatal area of upper epidermis in sepals (µm^2^). (**B**) Stomatal area of lower epidermis in sepals (µm^2^). (**C**) Stomatal area of upper epidermis in petals (µm^2^). (**D**) Stomatal area of lower epidermis in petals (µm^2^). (**E**) Vein density in sepals (mm^−1^). (**F**) Vein density in petals (mm^−1^). (**G**) Thickness of upper epidermis in sepals (µm). (**H**) Thickness of upper epidermis in petals (µm). (**I**) Thickness of upper epidermis in labellum (µm). (**J**) Thickness of lower epidermis in sepals (µm). (**K**) Thickness of lower epidermis in petals (µm). (**L**) Thickness of lower epidermis in labellum (µm). All data are presented as means ± SD. Different letters above bars within the same floral organ indicate significant differences among treatments (based on one-way ANOVA followed by Tukey’s post hoc test).

These traits are core indicators for evaluating floral water storage capacity. Progeny exhibited superiority over one or both parents in several indices, indicating significantly improved water storage capacity compared with the parental lines. From the perspective of water supply capacity (Appendix B Figure A3), vessel area of petals in progeny showed no significant difference from the female parent, but was significantly higher than that of the male parent (*p* = 0.044). Vascular bundle area of petals in progeny showed no significant difference from the female parent, and was also significantly higher than that of the male parent (*p* = 0.043). However, the vascular bundle area of sepals, vascular bundle area of labella, and the sieve tube area of sepals in progeny showed no significant difference from the male parent, but were significantly lower than those of the female parent (*p* = 0.005, *p* = 0.002, *p* = 0.003). These traits are key indicators reflecting floral water transport capacity. Overall, the female parent was superior to the progeny and the male parent in core water transport traits. There were no significant differences in floral vein density between parents and progeny (*p* > 0.05) (Appendix B Figure A4).

### 2.4. Differences in High-Temperature Tolerance Ability

**Figure 6:**
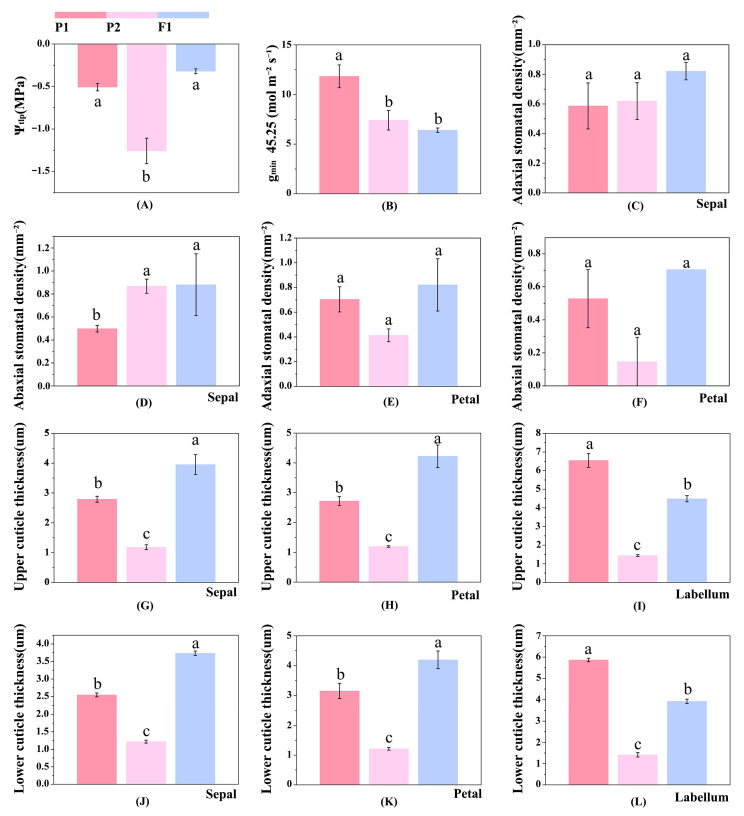
Analysis of differences in high-temperature tolerance between parental lines and progeny. (**A**) Water potential at turgor loss point (Ψ_tlp_, MPa). (**B**) Minimum diffusive conductance at 45.25 °C (g_min_ 45.25, mol m^−2^ s^−1^). (**C**) Stomatal density of the upper epidermis of sepals (mm^−2^). (**D**) Stomatal density of the lower epidermis of sepals (mm^−2^). (**E**) Stomatal density of the upper epidermis of petals (mm^−2^). (**F**) Stomatal density of the lower epidermis of petals (mm^−2^). (**G**) Cuticle thickness of the upper epidermis of sepals (μm). (**H**) Cuticle thickness of the upper epidermis of petals (μm). (**I**) Cuticle thickness of the upper epidermis of the labellum (μm). (**J**) Cuticle thickness of the lower epidermis of sepals (μm). (**K**) Cuticle thickness of the lower epidermis of petals (μm). (**L**) Cuticle thickness of the lower epidermis of the labellum (μm). All data are presented as means ± SD. Different letters above bars within the same floral organ indicate significant differences between treatments (*p* < 0.05, based on one-way ANOVA followed by Tukey’s post hoc test).

For stomatal density related to transpirational cooling, significant differences between the female parent and progeny were only detected in abaxial stomatal density of sepals (*p* = 0.024), with no significant differences in other floral organs (Figure 6). Overall, the female parent had fewer stomata and relatively weaker transpirational cooling capacity. In addition, adaxial and abaxial cuticle thickness of sepals and petals in progeny were significantly higher than those of both parents. Thickened cuticle can effectively reduce epidermal water transpiration and improve water retention under high temperature. For adaxial and abaxial cuticle thickness of labella, the male parent performed better than progeny and the female parent.

**Figure 7:**
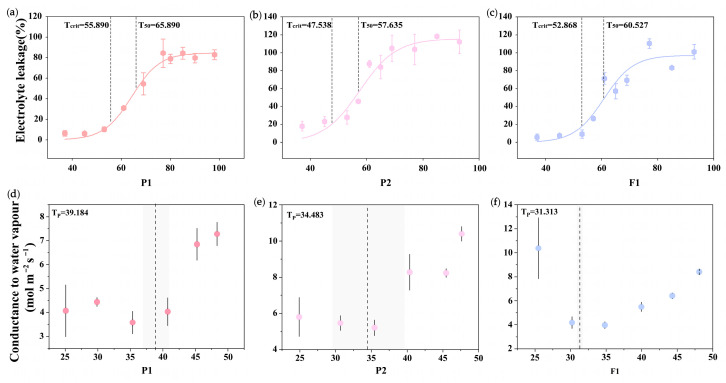
Analysis of electrolyte leakage and water vapor conductance in parental lines and their progeny. Panels (**a**–**c**) show electrolyte leakage data fitted with a sigmoidal function. Vertical dashed lines mark the critical thermal damage temperature (T_crit_, °C) and the high-temperature LT_50_ (T_50_, °C) for P1, P2, and F1. Panels (**d**–**f**) present water vapor conductance measured from 25 °C to 50 °C. In each panel, the vertical dashed line denotes the phase transition temperature (T_p_), and the corresponding value is given in the upper left corner. Error bars indicate the standard deviation (SD). Shaded gray regions indicate the temperature range surrounding T_p_.

The minimum diffusive conductance at 45.25 °C, a key indicator of water loss, showed no significant difference between the female parent and progeny, but both were significantly lower than that of the male parent (*p* = 0.012). The turgor loss point water potential (Ψ_tlp_) showed no significant difference between the male parent and progeny, but was significantly superior to that of the female parent (*p* < 0.001). In Figure 7, T_50_ (65.890, 60.527) and T_crit_ (55.890, 52.868) of the male parent and progeny were higher than T_50_ (57.635) and T_crit_ (47.538) of the female parent. The T_p_ value of the male parent (39.184) was significantly higher than those of the female parent (34.483) and progeny (31.313).

## 3. Discussion

### 3.1. Genetic Diversity Analysis of Parental Lines and Hybrid Progeny

Inter-primer binding site (iPBS) amplification, a multi-locus molecular marker technique based on retrotransposons, has the advantages of high efficiency, rapidity, and low cost, and has been widely used in studies related to diverse plant species [29]. To date, this technique has been widely applied in Orchidaceae research, but its application in *Vanda* remains relatively limited. In this study, iPBS markers were used to investigate the genetic characteristics of the male parent *P.* ‘Hetty Henderson’, the female parent *V. lamellata* var. *Boxallii* Rchb., and their hybrid progeny, so as to verify the applicability of this technique in cultivar identification, genetic diversity evaluation, and genetic relationship analysis of *Vanda* germplasm.

The results showed that the average polymorphism ratio of the six screened primers reached 92.98%, ranging from 85.71% to 100%, indicating abundant genetic diversity within this hybrid combination and confirming the high efficiency of iPBS markers compared with other molecular markers. The average polymorphism rate obtained in this study was similar to that reported in hybrid progeny identification of *Dendrobium* using iPBS markers [17], and higher than the average polymorphism ratio of SSR markers (85.91%) [30], as well as those of RAPD (86.50%) and ISSR (89.06%) markers [31].

Polymorphism information content (PIC) serves as a reliable index for evaluating marker polymorphism [32]. In this study, the PIC values of iPBS markers ranged from 0.339 to 0.475, with an average of 0.413, which was notably higher than the average PIC values of SSR markers reported in *Paphiopedilum* (0.1197 and 0.3097) [33]. Furthermore, the average Shannon’s information diversity index (I) of iPBS markers was 0.443, and the genetic similarity coefficients ranged from 0.460 to 0.965. These results further confirm the high efficiency and reliability of iPBS markers in detecting genetic diversity in *Vanda*, and demonstrate the abundant genetic variation among the tested accessions, supporting the selection of superior hybrid progeny for further cross-breeding.

### 3.2. Phenotypic Diversity Between Parental Lines and Hybrid Progeny

Differences in optical signals (reflectance and transmittance) among flowers of different colors arise from distinct combinations of pigment absorption properties and scattering structures [34]. Previous studies have shown that spectral variations in the 520–680 nm region are related to the absorption of green light by pigments such as chlorophyll and anthocyanins, whereas differences in longer wavelengths (e.g., 715–1000 nm) are mainly determined by the internal cellular structure of plant organs [35,36]. In the present study, the sepals of *Vanda* were darker than the petals and exhibited an extra characteristic valley at 650–700 nm compared with the petals, suggesting differentiation in pigment composition or cellular structure between the two organs. This may result from enhanced absorption of 650–700 nm light by sepals or reduced reflectance in this band due to cellular arrangement, forming an organ-specific spectral signature.

MARI has been verified to be strongly correlated with anthocyanin content in ray florets of chrysanthemum [20], and its capacity to characterize anthocyanin-mediated floral coloration shows certain universality across different species. In this study, MARI values differed significantly among *Vanda* accessions, and sepal MARI was significantly higher than petal MARI in the same individual (Figure 4a,d). Combined with the established relationship between MARI and anthocyanins, these results preliminarily indicate that anthocyanin-related optical characteristics are stronger in sepals than in petals. This is consistent with the findings reported by Narbona et al. (2018), who indicated that floral color polymorphism predominantly arises from anthocyanin-based pigmentation [37].

Our multi-dimensional cluster analysis based on iPBS molecular markers, morphological data, and floral spectral data revealed genetic and phenotypic associations in *Vanda* germplasm. Genetic, morphological, and spectral clustering all exhibited a consistent core pattern: the male parent formed an independent cluster, while the female parent and most hybrid progeny clustered together. This phenomenon has been observed in the hybrid progeny of many plant taxa. For example, maternal inheritance was also detected in intergeneric hybrids of *Phalaenopsis* based on genetic analysis of the ndhE gene fragment, indicating that traits of F_1_ progeny are generally biased toward the maternal parent [38]. Although morphological clustering was not fully consistent with genetic clustering, the female parent still dominated the morphological phenotypes of most progeny, reflecting the complexity of genetic regulation underlying phenotypic traits.

**Figure A5:**
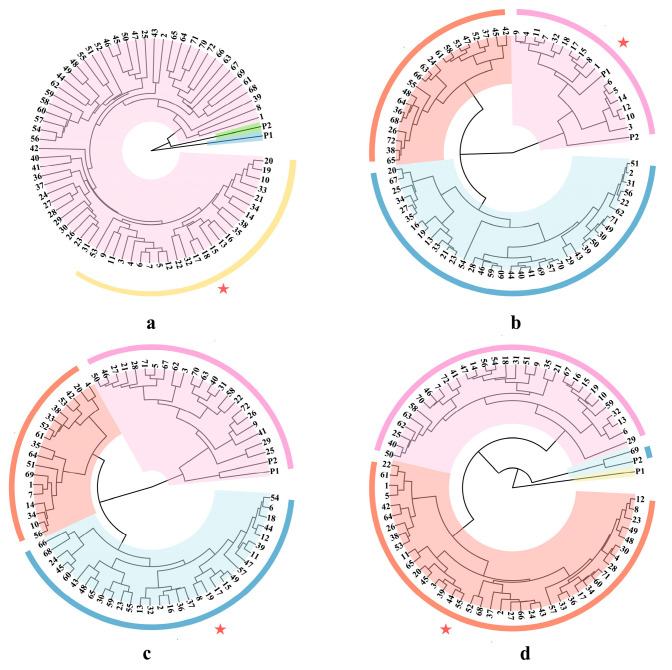
All clustering analysis results were plotted using the ggplot2 and ggtree packages in R language (version 4.5.0), with details as follows: (**a**) A circular clustering tree constructed based on DICE similarity coefficients calculated by NTSYS-pc 2.10e, reflecting the genetic similarity among samples; (**b**–**d**) represent clustering analysis plots based on Euclidean distance, where (**b**) corresponds to continuous morphological data, and (**c**,**d**) correspond to reflectance spectral data of floral organs (petals and sepals), respectively, aiming to resolve clustering differences among samples at the morphological and spectral phenotypic levels. The pentagram markers in the figures indicate the key distribution positions of progeny lines numbered 1 to 20, enabling intuitive identification of the population aggregation characteristics of this progeny group across different clustering dimensions. In different clustering diagrams, different colors represent distinct subgroups.

**Figure A6:**
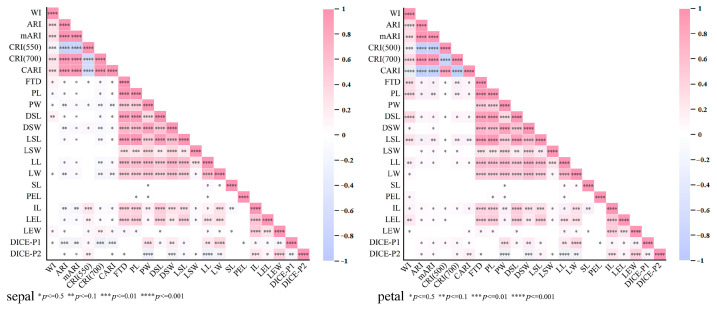
Correlation heatmaps of floral traits in petals (**left**) and sepals (**right**). Pearson correlation coefficient matrices among floral traits in sepals (**left**) and petal (**right**). Color intensity represents the strength of correlation: red indicates positive correlation, blue indicates negative correlation, and white indicates no correlation. Significance levels are marked as follows: * *p* ≤ 0.05; ** *p* ≤ 0.01; *** *p* ≤ 0.01; **** *p* ≤ 0.001.

The 1–20 morphologically similar accessions screened in the preliminary experiment showed high clustering consistency at the genetic (17 accessions in the same clade), morphological (15 accessions in the same cluster), and spectral phenotypic (11 accessions in the same clade) levels, indicating a relatively uniform genetic background and stable phenotypic characteristics within this group (Appendix B Figure A5). WI is derived from characteristic water absorption bands of plants (900 nm and 970 nm) and can quantitatively reflect tissue water content and status [39,40]. Petal WI was significantly positively correlated with FTD, PL, and SL (Appendix B Figure A6), and the dark-color ranges of sepal and petal WI within this group were highly consistent (Figure 4c,f). Only 6 out of 26 samples exhibited light-color petal and sepal WI values. Combined with the clustering results, it can be inferred that phenotypic characteristics of WI are coordinated with genetic background and morphological phenotypes. This supports the conclusion that spectral indices can link genetic background with metabolic phenotypes [41] and confirms that WI can serve as a stable quantitative indicator of floral physiological phenotypes to assist genetic and phenotypic association analysis.

### 3.3. Resistance Differences Between Parental Lines and Hybrid Progeny

Flowers adopt a low-carbon structural strategy: they invest minimally in carbon-based support tissues and rely on turgor-driven hydrostatic pressure for floral display. This makes floral water balance critical—stable water status from early development to anthesis supports flower growth, maintains pollinator-visible posture, ensures nectar secretion, and moderates temperature [42,43,44,45,46,47,48]. The most important water-balance strategy in flowers is reliance on internal water storage, reflected by higher hydraulic capacitance and relative water content. In this study, hydraulic and morphological traits were compared between parental lines and hybrid progeny based on genetic characteristics, and the water storage capacity of progeny was inferred to be superior to that of the parents to some extent (Figure 5). This advantage stemmed from both higher saturated water content (SWC), hydraulic capacitance before turgor loss (C_FT_), and hydraulic capacitance at turgor loss point (C_TLP_) compared with the male parent, and a higher apoplastic water fraction (a_f_) compared with the female parent. Previous studies suggested that the labellum requires high water storage capacity to maintain cell expansion and exhibits faster water loss at maturity than in the bud stage, whereas petals show the lowest water loss rate during anthesis [49]. In the present study, increased petal and sepal thickness in progeny, together with lower labellum thickness compared with the male parent, further indicated reduced water loss in hybrid progeny.

Early studies proposed that water supply to floral organs might rely primarily on the phloem, yet the phloem contributes insignificantly to the overall water balance of floral organs. Coupled with the inherently weak water retention capacity of floral organs, water supply thus remains centered on the xylem [44,45,48,50,51]. In the present study, the female parent exhibited superior performance in key water transport traits (vessel area and total vascular bundle area), which is hypothesized to reflect a functional trade-off with the differences in water storage capacity between parental lines and hybrid progeny: if the floral water storage capacity of the female parent was significantly lower than that of the progeny, it might compensate for the insufficient water storage by enhancing the water transport system (enlarging vessel and vascular bundle areas), so as to ensure the sustained water demand for floral growth and development. This is consistent with the conclusion proposed by Ke et al. [52] that flowers exhibit a trade-off between hydraulic safety and hydraulic efficiency, albeit with potential differences in floral adaptive strategies. Such trade-offs between different evolutionary clades or between parental lines and progeny may be achieved through divergent pathways, which reflect the diversity of water adaptation strategies in floral organs.

High temperature easily causes structural damage to floral organs and shortens flower longevity, thus reducing ornamental value. Therefore, quantifying the high-temperature tolerance of floral organs in hybrid progeny is of great importance. The heat-induced semi-lethal temperature (T_50_) is a key parameter for evaluating floral high-temperature tolerance, which directly reflects thermotolerance and is closely related to cell membrane stability [53,54]. The male parent exhibited superior adaptation to high temperature compared with the female parent and progeny: it possessed stronger heat resistance, with higher critical heat injury temperature (T_crit_) and T_50_ values than the female parent (Figure 7).

Phase transition temperature (T_p_) is another key indicator of floral heat tolerance. When ambient temperature exceeds a species-specific threshold, residual conductance (g_min_) of flowers increases significantly [55,56]. This threshold is defined as T_p_, which essentially represents the critical temperature at which cuticular permeability increases due to structural changes in cuticular waxes [57]. In the present study, the T_p_ value of the male parent (Figure 7) indicated superior cell membrane thermostability compared with the female parent and progeny, which was further supported by its stronger high-temperature tolerance. The male parent also exhibited significantly better water loss defense, such as higher minimum diffusive conductance at 45.25 °C, and thicker cuticles that further enhanced water retention and effectively reduced water loss under high temperature.

## 4. Materials and Methods

### 4.1. Plant Materials

Experimental materials were collected from September 2024 through December 2025 in the Orchid Germplasm Nursery at the Flower Research Institute, Guangxi Academy of Agricultural Sciences. Plant materials included the parental genotypes *P.* ‘Hetty Henderson’ and *V. lamellata* var. *Boxallii* Rchb., together with 72 hybrid progeny lines (numbered 1–72). Representative images of the male parent and selected progeny are presented in Figure 2. All plants were cultivated under stabilized conditions: 25–30 °C, 70–90% relative humidity, and 40% sunlight using shade nets throughout the growing period. For all measurements, uniformly developed flowers at 3–5 days post-anthesis and healthy, fully expanded young leaves were sampled from parental and hybrid plants to minimize phenological differences and seasonal environmental biases (Progeny flower year round, and all measurements were collected from September to December to minimize seasonal confounding effects.)

### 4.2. DNA Extraction and iPBS PCR Amplification

Young and healthy leaves were collected from parental lines and progeny lines, placed in sealed bags, labeled, stored in an ice box, and immediately transported to the laboratory. The samples were rapidly frozen in liquid nitrogen. Genomic DNA was extracted using an EasyPure^®^ Genomic DNA Kit with a desktop centrifuge (H1650-W, Cence, Changsha, China) and an electric thermostatic water bath (HWS-26, YIHENG, Shanghai, China). DNA integrity was examined by 2.0% agarose gel electrophoresis, and DNA concentration and purity were determined using a UV-visible spectrophotometer (UV-5500 PC, Metash Instruments, Shanghai, China). DNA samples were diluted to 20 ng/μL with TE buffer and stored at −20 °C until use.

PCR amplification was performed using a LifeECO thermal cycler (TC-96/G/H(b)C, Bioer, Hangzhou, China), an electrophoresis system (DYY-6D, LIUYI, Beijing, China), and an automatic gel imaging system (GenoSens 2000, Clinx, Shanghai, China). A total of 83 iPBS primers published by Kalendar et al. (2010) were initially screened using five randomly selected DNA samples [58]. Primers producing clear, stable, and highly polymorphic bands were selected for iPBS-PCR amplification. The optimal reaction system was determined based on the optimized iPBS protocol for *Dendrobium* described by Zhao et al. (2023) [59].

PCR amplification was carried out in a total volume of 20 μL, containing 10 μL of 2×EasyTaq^®^ PCR SuperMix, 8 μL of sterile deionized water, 1 μL of primer, and 1 μL of template DNA. The amplification program was set as follows: pre-denaturation at 94 °C for 4 min; 35 cycles of denaturation at 94 °C for 1 min, annealing at 50 °C (adjusted for different primers) for 45 s, and extension at 72 °C for 2 min; and a final extension at 72 °C for 10 min. PCR products were stored at 4 °C until electrophoresis analysis.

### 4.3. Measurement of Flower Morphological Traits

Floral morphological traits were measured using a digital vernier caliper on uniformly sampled flowers, as described above. The trait measurement and evaluation were strictly conducted in accordance with the industrial standard NY/T 2758-2015 Guidelines for the Conduct of Tests for Distinctness, Uniformity and Stability—New Plant Varieties: *Dendrobium*, issued by the Ministry of Agriculture and Rural Affairs of the People’s Republic of China in 2015. Three biological replicates were performed for each trait, and the final results are presented as the mean values.

### 4.4. Measurement of Petal and Sepal Spectral Phenotypes

To minimize environmental interference, all spectral measurements were taken in situ at the Orchid Germplasm Nursery on clear sunny mornings between 9:00 and 12:00. The transmittance and reflectance spectra of petals and sepals from parental and progeny lines were measured using a portable leaf spectrometer (CI-710, CID Bio-Science, Logan, UT, USA). The instrument was connected to a computer via USB, powered on, and preheated for 10 min to stabilize before measurement. Three biological replicates were performed for each line, and the average reflectance values were used to calculate the spectral reflectance indices for petals and sepals (Table 4). Where Equations (1) and (2) are from [60], Equation (3) is from [61], Equations (4) and (5) are from [62], and Equation (6) is from [63].

The spectral indices were calculated as follows:

ARI = R^−1^*_λ_*_green_ − R^−1^*_λ_*_red edge_ (1)

MARI = (R^−1^*_λ_*_green_ − R^−1^_λred edge_)R_nir_ (2)

CARI = R_720_/R_521_ −1 (3)

CRI_550_ = (R_510_) − 1 − (R_550_) − 1 (4)

CRI_700_ = (R_510_) − 1 − (R_700_) − 1 (5)

WI = R_900_/R_970_ (6)

R: Spectral reflectance; *λ*_red edge_: Average spectral reflectance in the red edge region (690–710 nm); *λ*_green_: Average spectral reflectance in the green region (540–560 nm). Equations (1) and (2) are from [60], Equation (3) is from [61], Equations (4) and (5) are from [62], and Equation (6) is from [63].

Based on the above comprehensive analyses of molecular markers, morphology and spectroscopy, 20 morphologically similar accessions (No. 1–20) were preliminarily screened in the preliminary experiment of this study. Among the star-marked individuals, 17 accessions were clustered into the same clade in the genetic dendrogram, 15 formed an independent group in the morphological clustering, and 11 belonged to the same branch in the floral organ spectral clustering (Appendix B Figure A5). This population showed high consistency in clustering patterns at the genetic, morphological and spectral phenotypic levels, indicating relatively uniform genetic background and stable phenotypic traits, making it suitable for subsequent studies on resistance differences.

### 4.5. Anatomic Structure of the Perianth

Perianth cross-sections were prepared using a modified paraffin sectioning method to observe and measure perianth thickness, cuticle thickness, xylem area, phloem area, maximum vessel area, and maximum sieve tube area. Samples were dehydrated in a graded ethanol series, cleared with graded xylene solutions, infiltrated with low- and high-melting-point paraffin, then embedded and sectioned into 10 μm slices. After dewaxing, sections were stained with safranin and fast green and mounted in neutral balsam. Microscopic observation and imaging were conducted using a Leica DM 3000 optical microscope. To measure floral vein density, 3–6 fully open flowers per treatment group were randomly selected, separated into parts, and fixed in FAA fixative for over 30 days for fixation and discoloration. After the perianths turned transparent, they were dried and scanned with an HP Scanjet G3110 scanner. Vein lengths (longitudinal, transverse, open) and perianth area were measured via ImageJ 1.54p, and vein density (mm mm^−2^) was calculated as vein length/perianth area.

### 4.6. Measurement of Pressure–Volume Parameters of Flowers

Pressure-volume curves were generated using standard methods: bulk water potential was repeatedly measured with a pressure chamber (0.01 MPa resolution; PMS Instruments, Albany, OR, USA), and mass was recorded to determine the relationship between water potential and content. For each treatment, 3–5 fully bloomed flowers were randomly selected. Before measurement, whole plants were moved to the laboratory to reduce post-detachment water loss effects, and the pressure chamber was humidified with wet paper towels to prevent evaporation during measurements. Balancing pressure was determined by slowly increasing chamber pressure until water appeared at the cut pedicel/petiole, then reducing pressure to ambient. The specimen was immediately weighed (0.0001 g resolution). Post-measurement, total flower surface area was measured with a leaf area meter (Li-3000A, LI-COR, Lincoln, NE, USA), followed by drying at 75 °C for at least 72 h to constant weight for dry mass determination. From pressure-volume curves, we calculated hydraulic traits (Table 4).

### 4.7. Flower Thermotolerance

Flower cell membrane thermostability was determined using the ion leakage method. Fully open flowers were collected and cut into fragments, and 0.1–0.15 g of samples were vacuum-infiltrated in 35 mL deionized water for 15 min. Initial electrical conductivity (C_n_) was measured at 25 °C. Samples were then heated in a water bath from 37 to 93 °C at 7 °C intervals, incubated for 20 min at each temperature, and cooled to measure conductivity (C_i_). After boiling for 20 min and cooling, total conductivity (C_t_) was determined. Electrolyte leakage (EL_i_, %) was calculated as [64]:

EL_i_ (%) = (C_i_ − C_n_)/(C_t_ − C_n_) × 100. (7)

The electrolyte leakage curve was plotted against temperature, and a three-parameter sigmoid model was fitted using the nls. multstart package in R 4.5.0 to estimate the critical temperature (T_crit_) corresponding to irreversible heat damage in flowers.

### 4.8. Determination of Minimum Epidermal Surface Conductance (g_min_) and Temperature of Phase Transition (T_p_) of Flowers

For each treatment, 3–6 fully open flowers were randomly selected to determine the minimum epidermal surface conductance (g_min_) under stable laboratory temperature and humidity. Floral organs were separated and weighed for fresh mass, and petal wounds were sealed with wax. Samples were exposed to steady airflow from a fan and weighed at 10 min intervals, with temperature and relative humidity recorded for 8–10 repeated measurements. The projected area of each organ was measured using a Li-3000A leaf area meter. Samples were then oven-dried at 75 °C for more than 24 h to a constant dry mass.

The g_min_ was calculated as:

g_min_ = W_L_·P_atm_/VPD (8)

where W_L_ is the water loss rate normalized by surface area or dry mass, P_atm_ is atmospheric pressure (101.3 kPa), and VPD is the vapor pressure deficit. To examine temperature effects and phase transition temperature (T_p_) among floral organs, g_min_ was further measured at 25, 30, 35, 40, 45, and 50 °C in a growth chamber. The temperature dependence of g_min_ was analyzed using an Arrhenius plot, in which the natural logarithm of g_min_ was plotted against the inverse of absolute temperature.

### 4.9. Methods for Constructing Clustering Trees

In the present study, a rectangular phylogenetic tree (Figure 2) was generated by constructing a genetic similarity coefficient matrix and performing clustering analysis using the Dice function within the SIMQUAL module of the NTSYS-pc 2.10e. All circular clustering trees were constructed in R software (version 4.5.0): clustering trees were plotted using the ggplot2 and ggtree packages based on DICE similarity coefficients calculated by NTSYS-pc 2.10e and Euclidean distance. To meet the graphical standards for academic journal publication, Adobe Photoshop CC 2020 was finally used for further optimization and typesetting of the images, including color calibration, legend adjustment, text modification, and vector graphic optimization, without altering the original data distribution and clustering results.

## 5. Conclusions

In this study, inter-primer binding site (iPBS) molecular markers, morphological markers, and spectral technology were combined to identify intergeneric hybrid progenies between *Papilionanthe* and *Vanda*. The results revealed high genetic diversity within the progeny population. Genetic clustering, morphological clustering, and spectral phenotyping consistently indicated that hybrid progenies were genetically closer to the female parent than to the male parent. Morphological identification showed that progeny phenotypes integrated traits from both parents, while spectral phenotyping revealed that the clustering patterns of phenotypic indices were highly consistent with the genetic clades defined by iPBS markers. This suggests that floral spectral phenotypic variation in *Vanda* is partly associated with the genetic background revealed by iPBS analysis.

Comparative analysis of stress resistance showed that progenies exhibited stronger water storage capacity than the parental lines, whereas the female parent performed better in key water-transport traits, implying a potential functional trade-off between water storage and water transport among parents and progenies. The male parent displayed higher critical heat injury temperature (T_crit_) and heat-induced semi-lethal temperature (T_50_), as well as stronger water retention and thicker cuticles, indicating significantly superior high-temperature adaptation relative to the female parent and progenies.

Overall, systematic analysis of parent–progeny relationships at the molecular, spectral phenotypic, morphological, and stress-resistance levels helps clarify the genetic regulatory mechanisms underlying trait variation in *Vanda* hybrids. This study also provides efficient technical support and a theoretical basis for early and precise target trait selection in orchid breeding.

## Figures and Tables

**Table 1:** Amplification results of the six interprimer binding site (iPBS) primers.

Primer	Sequences (5′-3′)	AT (°C)	TB	PB	PPB (%)	PIC
2270	ACCTGGCGTGCCA	50	14	12	85.71	0.339
2224	ATCCTGGCAATGGAACCA	50	16	14	87.50	0.392
2232	AGAGAGGCTCGGATACCA	51	19	19	100	0.475
2238	ACCTAGCTCATGATGCCA	50	16	15	93.75	0.417
2243	AGTCAGGCTCTGTTACCA	48	14	14	100	0.424
2251	GAACAGGCGATGATACCA	48	11	10	90.91	0.431
Total			90	84		2.478
Average			15	14	92.98	0.413
Standard deviation						0.731

**Table 2:** Genetic diversity indices of parental lines and 72 progeny accessions.

Simple Size	Na	Ne	He	I
74	1.933 ± 0.251	1.489 ± 0.333	0.293 ± 0.166	0.443 ± 0.224

Na, Observed number of alleles; Ne, Effective number of alleles; He, Nei’s gene diversity index; I, Shannon’s Information index.

**Table 3:** Genetic Performance of Major Ornamental Traits in Parents and Progeny Lines (Sample Nos. 1–20).

			Progeny						
Plant Traits	Female Parent	Male Parent	Mean	Coefficient of Variation (CV)%	Range	Mid-Parent Heterosis (%)	Better Than Both Parents (%)	Intermediate Between Parents (%)	Worse Than Both Parents (%)
FTD	5.81	4.57	7.13 ± 0.482	6.77	5.68–7.13	37.29	95.00	5.00	0.00
DSL	2.89	2.30	3.45 ± 0.205	5.96	2.84–3.71	33.13	95.00	5.00	0.00
DSW	2.30	1.02	1.42 ± 0.116	8.15	1.17–1.59	7.66	0.00	100.00	0.00
LSL	2.73	1.80	3.20 ± 0.200	6.24	2.80–3.62	17.47	100.00	0.00	0.00
LSW	1.73	1.30	1.62 ± 0.157	9.83	1.29–1.90	6.74	10.00	90.00	0.00
PL	2.77	2.25	3.40 ± 0.203	6.00	2.85–3.76	35.47	100.00	0.00	0.00
PW	2.01	0.98	1.34 ± 0.165	12.30	1.10–1.66	−10.18	0.00	100.00	0.00
LL	2.12	1.27	1.92 ± 0.085	4.44	1.71–2.03	13.45	0.00	100.00	0.00
LW	1.34	0.51	0.92 ± 0.126	13.75	0.73–1.27	−0.49	0.00	100.00	0.00
SL	0.55	0.60	1.49 ± 0.130	8.73	1.18–1.65	160.09	100.00	0.00	0.00
PedL	3.25	5.40	4.94 ± 0.408	8.26	4.17–5.93	14.27	15.00	85.00	0.00
IL	33.06	53.67	35.34 ± 6.40	18.12	21.20–45.00	−18.52	0.00	75.00	25.00
LEL	21.95	23.63	29.68 ± 2.45	8.27	25.20–32.97	30.22	100.00	0.00	0.00
LEW	0.43	1.64	0.86 ± 0.076	8.75	0.64–0.97	−16.42	0.00	100.00	0.00

**Table 4:** Spectral Indices Measured in the Present Study and Corresponding Indicators Associated with Resistance Differences.

Symbol	Definition	Units
ARI	Anthocyanin Reflectance Index	-
MARI	Modified Anthocyanin Reflectance Index	-
CARI	Carotenoid Index	-
CRI_550_	Carotenoid Reflectance Index	-
CRI_700_	Carotenoid Reflectance Index	-
WI	Water Index	-
PT	Perianth thickness	μm
C_FT_	hydraulic capacitance before turgor loss point	mol m^−2^ MPa^−1^
C_TLP_	hydraulic capacitance at turgor loss point	mol m^−2^ MPa^−1^
SWC	Saturated water content	g g^−1^
a_f_	Apoplastic water fraction	%
g_min_	Minimum epidermal surface conductance	mmol m^−2^ s^−1^
Ψ_tlp_	Water potential at turgor loss point	MPa
ε	Cell elastic modulus	MPa
T_crit_	Critical temperature at which the flower undergo irreversible heat damage	°C
T_50_	Temperature leading to 50% leakage of electrolyte	°C
SS	Stomatal size	μm^2^
CT	Cuticle thickness	μm
VA	Vascular bundle area	μm^2^
XA	Xylem area	μm^2^
PA	Phloem area	μm^2^
FVD	Flower vein density	μm^2^

## Data Availability

The authors fully support and agree to share the research data generated in this study in accordance with the MDPI Research Data Policies. All raw and processed data supporting the findings of this article are available from the corresponding author upon reasonable request via official email. The data will be provided to qualified researchers for academic and non-commercial use only, to ensure the proper replication and verification of the results presented herein.

## Appendix A

**Table A1:** Differences in Spectral Indices of Petals and Calyxes between Parents and Progeny.

	Petal			Sepal		
	Female Parent	Male Parent	Progeny	Female Parent	Male Parent	Progeny
ARI	0.791	−0.025	3.446	0.346	8.174	6.550
mARI	0.184	0.209	0.554	0.201	3.333	0.039
CARI	0.300	−0.019	1.345	0.105	3.004	2.615
CRI_550_	−0.377	−0.003	−1.437	−0.193	1.462	−2.273
CRI_700_	0.444	−0.041	2.280	0.170	8.225	4.831
WI	1.013	0.990	1.063	1.023	1.119	1.101
